# Healthcare Management in Cardio-Oncology, Clinical Strategies and Future Perspectives: A Narrative Review

**DOI:** 10.3390/healthcare13202599

**Published:** 2025-10-15

**Authors:** Vincenzo Quagliariello, Massimiliano Berretta, Fabrizio Maurea, Matteo Barbato, Andrea Paccone, Martina Iovine, Alfredo Mauriello, Celeste Fonderico, Domenico Gabrielli, Andrea Camerini, Carmine Riccio, Marino Scherillo, Stefano Oliva, Maria Laura Canale, Nicola Maurea

**Affiliations:** 1 Division of Cardiology, Istituto Nazionale Tumori-IRCCS-Fondazione G. Pascale of Naples, 80131 Naples, Italy; matteo.barbato@istitutotumori.na.it (M.B.); andreapaccone@gmail.com (A.P.); mart.iovine@gmail.com (M.I.); alfredo.mauriello@istitutotumori.na.it (A.M.); celeste.fonderico@istitutotumori.na.it (C.F.); n.maurea@istitutotumori.na.it (N.M.); 2 Department of Clinical and Experimental Medicine, University of Messina, 98124 Messina, Italy; berrettama@gmail.com; 3 Department of Medical Oncology, University Federico II of Naples, 80138 Naples, Italy; 4 UOC Cardiologia, Dipartimento Cardio-Toraco-Vascolare, Azienda Ospedaliera San Camillo Forlanini, Roma—Fondazione per il Tuo Cuore—Heart Care Foundation, 50121 Firenze, Italy; dgabrielli@scamilloforlanini.rm.it; 5 Medical Oncology, Versilia Hospital, Azienda USL Toscana Nord-Ovest, 55041 Lido di Camaiore, Italy; andrea.camerini@uslnordovest.toscana.it; 6 UOSD Follow-up del Paziente Post-Acuto, Dipartimento Cardio-Vascolare, AORN Sant’Anna e San Sebastiano, 81100 Caserta, Italy; carminericcio8@gmail.com; 7 Cardiologia Interventistica e UTIC, A.O. San Pio, Presidio Ospedaliero Gaetano Rummo, 82100 Benevento, Italy; marino.scherillo@gmail.com; 8 UOSD Cardiologia di Interesse Oncologico-IRCCS-Istituto Tumori “Giovanni Paolo II”, 70124 Bari, Italy; s.oliva@oncologico.bari.it; 9 Cardiology Department, Versilia Hospital, Azienda USL Toscana Nord-Ovest, 55041 Lido di Camaiore, Italy; marialaura.canale@uslnordovest.toscana.it

**Keywords:** cancer, cardio-oncology, management, healthcare, primary prevention, secondary prevention, diagnosis

## Abstract

The growing overlap between cardiovascular disease and cancer has made cardio-oncology a key subspecialty in modern oncology care. Improved cancer survival has increased the burden of therapy-related cardiovascular complications, including heart failure, arrhythmias, ischemic events, and vascular toxicity, driven by oxidative stress, endothelial dysfunction, immune-mediated injury, and metabolic vulnerability. Effective management requires a continuum-of-care approach, integrating baseline risk assessment, biomarker- and imaging-guided surveillance, and timely cardioprotective therapy without compromising cancer treatment. Key strategies include validated risk scores (HFA/ICOS, Mayo), early detection of subclinical dysfunction via troponin, natriuretic peptides, and strain imaging, and proactive cardioprotective agents such as ACE inhibitors, beta-blockers, SGLT2 inhibitors, and statins in high-risk patients. This narrative review summarizes risk-stratification models, structured care pathways, and multidisciplinary hub-and-spoke networks linking specialized centers with community oncology services. It emphasizes modifiable cardiometabolic factors, obesity, insulin resistance, NAFLD, sarcopenia, and chronic inflammation, which heighten cardiotoxicity risk and should guide precision prevention and survivorship care. We also address emerging challenges, including the integration of digital health, tele-monitoring, and AI-based decision support, and the shift toward value-based reimbursement models, highlighting persistent barriers such as data privacy, infrastructure gaps, and inequitable access to specialized care.

## 1. Introduction

The rapid evolution of cancer therapies over the past two decades has markedly improved survival among oncology patients [1]. However, this progress has revealed a growing burden of treatment-related cardiovascular complications, increasingly relevant in long-term cancer care [2]. Cardio-oncology, an emerging subspecialty at the interface of cardiology and oncology, aims to preserve oncologic efficacy while preventing, detecting, and managing therapy-induced cardiovascular toxicity [3]. A broad range of agents contributes to cardiotoxicity. Traditional anthracyclines are well known for their dose-dependent cardiomyopathy and heart failure. Platinum-based compounds (e.g., cisplatin) and alkylating agents can cause vascular injury, leading to hypertension, thromboembolism, and accelerated atherosclerosis [4]. Among newer treatments, HER2-targeted therapies (e.g., trastuzumab, pertuzumab) often cause reversible left-ventricular dysfunction, especially when combined with anthracyclines [5]. VEGF inhibitors (e.g., bevacizumab, sunitinib, sorafenib) are linked to hypertension, thromboembolism, and heart failure via endothelial dysfunction [6], while BCR-ABL tyrosine kinase inhibitors (e.g., imatinib, dasatinib, ponatinib) may lead to pulmonary hypertension, arterial occlusion, and arrhythmias [7]. Moreover, proteasome inhibitors (e.g., bortezomib, carfilzomib) and immunomodulatory drugs (e.g., thalidomide, lenalidomide) can increase thromboembolic and ischemic risk [8]. Immune checkpoint inhibitors (e.g., nivolumab, pembrolizumab, ipilimumab) have transformed cancer care but carry the risk of immune-mediated myocarditis, pericarditis, and vasculitis, requiring rapid recognition and intervention [9]. In addition, hormonal therapies (e.g., aromatase inhibitors, androgen-deprivation therapy) increase cardiovascular risk, particularly in older patients with comorbidities [10]. Recognition of these risks has driven the publication of key international guidelines, including the 2022 European Society of Cardiology (ESC) Cardio-Oncology Guidelines, which offer comprehensive recommendations for risk assessment, surveillance, and management of therapy-related cardiovascular complications, and the American Society of Clinical Oncology (ASCO) guidance on survivorship and cardiotoxicity. Recent International Cardio-Oncology Society (ICOS) consensus statements further emphasize the need for multidisciplinary coordination, equitable access, and global harmonization of care. These frameworks contextualize the present review and highlight the importance of implementing guideline-based strategies across diverse healthcare systems. Beyond acute cardiotoxicity, the field must address long-term survivorship and late cardiovascular effects, especially in patients with shared risk factors such as hypertension, diabetes, dyslipidemia, and obesity [11]. From a healthcare management perspective, this complexity requires robust integrated care models. Cardio-oncology is most effective within a multidisciplinary framework involving cardiologists, oncologists, internists, radiologists, nurses, pharmacists, rehabilitation specialists, and patient navigators [12]. This team-based approach enables early risk identification, biomarker- and imaging-based surveillance, and coordinated therapeutic decisions that balance oncologic efficacy with cardiovascular safety (Table 1) [13].

Effective healthcare delivery in cardio-oncology also relies on the establishment of dedicated cardio-oncology units or services, embedded within cancer centers or cardiovascular institutions [14]. These units enable protocolized care pathways for pre-treatment risk stratification, in-treatment surveillance (e.g., echocardiography, troponin/BNP assays, ECG monitoring), and post-treatment cardiovascular follow-up [15]. Risk-adapted monitoring schedules and shared electronic health records are crucial tools for communication and continuity of care [16]. Moreover, health-system-level strategies must address resource allocation, quality metrics, patient access, and health equity. Many healthcare systems face the challenge of limited awareness, variable clinical expertise, and a lack of standardized guidelines, especially in community or rural settings. Reimbursement models and policy frameworks must evolve to support preventive cardiology in cancer care, value-based care delivery, and the incorporation of digital health technologies (e.g., telemedicine, AI-assisted diagnostics, remote monitoring) (Table 2).

This review will explore these clinical and systemic dimensions of cardio-oncology management, situating them within the context of recent global guidelines, and offering a comprehensive yet practice-oriented overview aimed at improving outcomes for the growing population of cancer patients and survivors with cardiovascular comorbidities.

## 2. Materials and Methods

This article is conceived as a narrative, clinically oriented review of current strategies for the management of cardiovascular risk in cancer patients and survivors. Its primary aim is to provide clinicians and health-system stakeholders with a pragmatic synthesis of evidence and expert guidance, rather than to produce a formal systematic review or meta-analysis. To ensure transparency in the selection of sources, we described a structured search strategy that guided, but did not determine, the narrative synthesis. A comprehensive search was conducted in PubMed/MEDLINE and EMBASE for articles published from January 2010 to September 2025, using combinations of the following keywords and Boolean operators: “cardio-oncology” OR “cardiotoxicity” AND (“risk stratification” OR “surveillance” OR “biomarkers” OR “imaging” OR “multidisciplinary care” OR “hub-and-spoke” OR “policy” OR “value-based care” OR “digital health”). The search was limited to English-language publications with available abstracts. Notably, we included clinical studies, prospective and retrospective cohorts, randomized controlled trials, meta-analyses, and relevant registry reports addressing cardiovascular complications of cancer therapies, clinical risk stratification, patient profiling, organizational models of cardio-oncology care, policy aspects, and digital-health solutions. Reference lists of relevant articles, guideline statements, and position papers were also screened to identify additional pertinent literature (Table 3).

We excluded narrative reviews, case reports, editorials, commentaries, experimental pre-clinical studies without direct clinical implications, and conference abstracts without full data. Risk-of-Bias Considerations: Although the review is not systematic and no pooled quantitative synthesis was performed, we assessed the quality of included evidence by considering key domains relevant to clinical studies:

- Patient selection (representativeness of study populations, inclusion/exclusion criteria);
- Outcome ascertainment and follow-up (definition and measurement of cardiotoxicity or clinical endpoints);
- Control for confounding (adjustment for baseline cardiovascular risk factors, treatment exposure, and competing risks).

This qualitative appraisal was informed by the Joanna Briggs Institute (JBI) Critical Appraisal Checklists for observational studies [17] and, when applicable, by the Cochrane Risk-of-Bias framework for randomized trials. These tools were used to guide judgment on the relative weight assigned to different types of evidence in the discussion.

Although we employed a reproducible search strategy for transparency, we did not adopt a full PRISMA methodology, including duplicate screening, formal scoring of risk of bias, or meta-analytic pooling, because of the heterogeneity of study designs (clinical trials, registries, organizational models, and policy analyses) and the practical focus on management and health-system implementation. A formal PRISMA-based systematic review was therefore considered inappropriate for the present purpose. To make this distinction explicit, we state that the search results were used to inform a structured narrative synthesis, not to support a systematic review. Wherever feasible, we specify in the text whether the conclusions derive from meta-analyses, multicenter studies, or smaller observational series, thereby enabling the reader to gauge the robustness of the evidence base.

## 3. Clinical Risk Stratification and Patient Profiling

Effective cardio-oncology management begins with early and precise risk stratification. Clinical risk prediction models must incorporate both baseline cardiovascular risk factors, such as hypertension, diabetes, dyslipidemia, and previous heart disease, and oncologic treatment-specific risk, including the cumulative dose and class of cardiotoxic agents. Tools such as the Heart Failure Association (HFA)/International Cardio-Oncology Society (ICOS) risk score and the Mayo Clinic cardio-oncology risk algorithm help categorize patients into low, intermediate, or high cardiotoxicity risk groups [18]. These models are further refined by integrating cardiac biomarkers (e.g., troponin, NT-proBNP), echocardiographic parameters (e.g., left ventricular ejection fraction [LVEF], global longitudinal strain [GLS]), and functional capacity assessments [19]. In pediatric populations and hematologic malignancies, stratification must account for long-term toxicity profiles and genetic predisposition. Personalized risk assessment guides surveillance intensity and informs oncologic decision-making, enabling clinicians to balance cardioprotection with effective cancer therapy [20].

**Figure 1:**
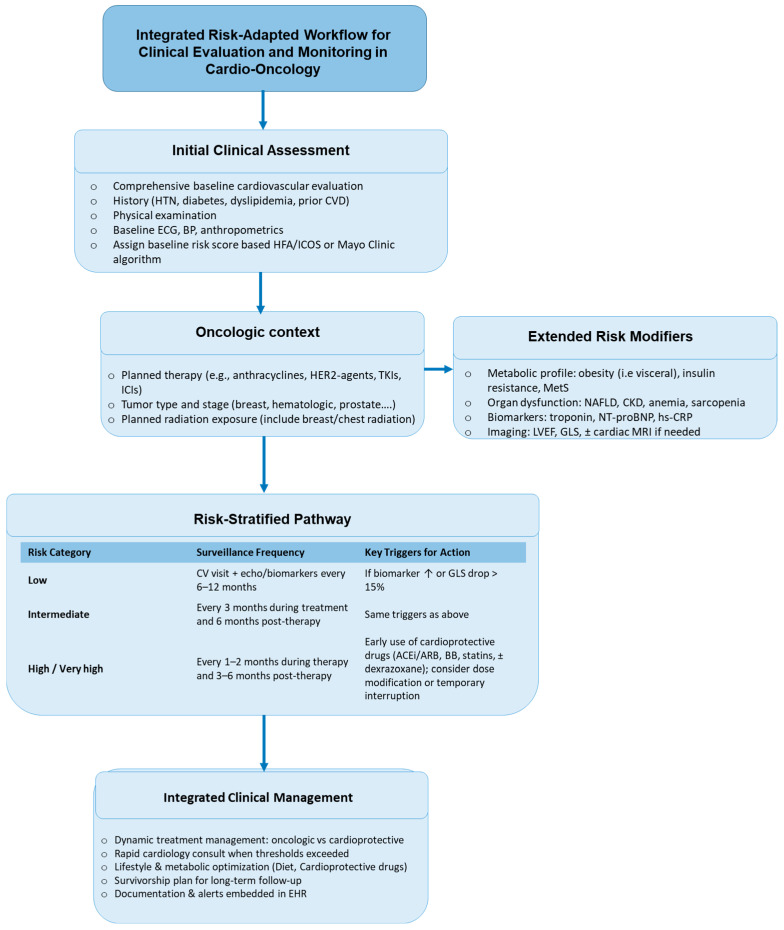
*Integrated risk-adapted workflow for clinical evaluation and monitoring in cardio-oncology*. The diagram summarizes a step-wise, clinically actionable pathway that integrates baseline cardiovascular assessment, oncologic treatment context, and extended risk modifiers (metabolic, organ-related, and imaging/biomarker data) to guide risk stratification and surveillance. Patients are categorized as low, intermediate, or high/very-high risk, which determines the recommended frequency of cardio-oncology visits and echocardiographic/biomarker monitoring. Specific thresholds—such as a ≥15% reduction in GLS, a ≥10-point fall in LVEF to <50%, or persistent elevation of troponin/NT-proBNP—trigger early cardioprotective interventions (ACE inhibitors/ARBs, β-blockers, statins, ± dexrazoxane, and, as indicated, SGLT2 inhibitors, sacubitril/valsartan, GLP-1 receptor agonists, or PCSK9 inhibitors) and multidisciplinary re-evaluation of oncologic therapy. The workflow emphasizes dynamic adjustment of treatment, early cardiology involvement, optimization of metabolic health, and long-term survivorship planning, supported by structured documentation and electronic health-record alerts.

However, a more comprehensive understanding of patient vulnerability in cardio-oncology must also include an evaluation of the cardiometabolic profile, which often plays a pivotal yet under-recognized role in mediating both baseline cardiovascular risk and therapy-induced toxicity [21]. Obesity, particularly visceral adiposity, has emerged as a key contributor to both cancer progression and cardiovascular dysfunction. Visceral fat promotes a pro-inflammatory state, increases oxidative stress, and secretes adipokines such as leptin and resistin, which impair endothelial function and drive myocardial remodeling [21]. Elevated leptin levels in particular have been linked to both tumor proliferation and left ventricular hypertrophy, posing a dual risk in cancer patients. Closely related is insulin resistance, which promotes arterial stiffness, impaired coronary flow reserve, and adverse myocardial metabolism [22]. It is often a central feature of the Metabolic Syndrome (MetS), a cluster of interrelated risk factors that include central obesity, hypertension, dyslipidemia (elevated triglycerides and reduced HDL), and impaired fasting glucose [23]. These metabolic abnormalities are commonly seen in patients with breast, prostate, colorectal, and hematologic malignancies and significantly increase the risk of cardiotoxicity from therapies such as anthracyclines, HER2-targeted therapies, and tyrosine kinase inhibitors (Figure 1). Notably, radiation therapy to the breast and mediastinum remains an essential component of treatment for early-stage and locally advanced breast cancer. However, exposure of the heart and great vessels to ionizing radiation carries a well-established risk of late-onset cardiovascular complications, including accelerated coronary artery disease, valvular thickening and dysfunction, restrictive cardiomyopathy, conduction abnormalities, and pericardial disease [21].

This table (Table 4) summarizes the major domains contributing to cardio-oncology risk assessment and illustrates how they can be integrated into validated tools such as the HFA/ICOS risk score and the Mayo Clinic algorithm. In addition to baseline cardiovascular comorbidities and oncologic treatment exposures, extended modifiers—including metabolic dysfunction (visceral obesity, insulin resistance, diabetes, NAFLD), sarcopenia, endocrine disorders, inflammatory burden, and impaired functional reserve—enhance cardiotoxicity risk and support a more personalized approach. Incorporation of dynamic biomarkers (troponin, NT-proBNP) and imaging parameters (LVEF, GLS) allows re-stratification during therapy and guides early introduction of cardioprotective strategies (e.g., ACE inhibitors/ARBs, β-blockers, statins, SGLT2 inhibitors, sacubitril/valsartan, GLP-1 receptor agonists, PCSK9 inhibitors, and, when appropriate, dexrazoxane). This integrated profiling approach links baseline risk factors to surveillance intensity and intervention triggers, enabling a clinically actionable workflow for cardio-oncology care.

The risk is dose-dependent and correlates with the mean heart dose (MHD) and the volume of cardiac structures within the radiation field. A landmark population-based study demonstrated an approximately 7% relative increase in the risk of major coronary events for each additional 1 Gy of mean heart dose [17]. Advances in modern radiotherapy techniques, including deep-inspiration breath hold, prone positioning, 3-D conformal radiotherapy, intensity-modulated radiotherapy (IMRT), and proton-beam therapy, have significantly reduced cardiac exposure compared with historical tangential-field methods [22,23]. Nevertheless, the potential for late cardiovascular sequelae persists, particularly in patients receiving concomitant cardiotoxic systemic therapies such as anthracyclines or HER2-targeted agents. Current ESC cardio-oncology guidelines (2022) and ASCO survivorship statements recommend baseline cardiovascular risk assessment and individualized long-term surveillance for breast cancer survivors who received left-sided or high-dose mediastinal radiation. Integration of radiation-dose parameters (e.g., MHD, dose to left anterior descending coronary artery) into the patient’s composite cardio-oncology risk profile facilitates tailored monitoring and preventive strategies, including lifestyle optimization, pharmacologic cardio protection where appropriate, and early referral for echocardiography or coronary imaging in high-risk survivors (Table 4).

Hyperglycemia and type 2 diabetes mellitus (T2DM) further exacerbate cardiotoxicity through mechanisms such as glycation end-product formation, mitochondrial dysfunction, and microvascular injury [24]. The presence of non-alcoholic fatty liver disease (NAFLD), often coexisting with MetS and insulin resistance, also warrants attention in cardio-oncology [25]. NAFLD is associated with systemic inflammation, endothelial dysfunction, and prothrombotic states, all of which contribute to increased cardiovascular risk. Moreover, hepatic dysfunction may alter drug pharmacokinetics and amplify toxicity. Given its high prevalence, NAFLD should be considered a silent enhancer of cardiotoxic risk in cancer patients, especially those undergoing polypharmacy or hepatically cleared treatments [26] (Table 4).

Recent quantitative analyses further underscore the clinical relevance of these metabolic modifiers. A meta-analysis of 13 prospective studies (>12,000 patients) reported that obesity was associated with a 1.6-fold higher risk of anthracycline-related left-ventricular dysfunction, independent of cumulative dose and baseline LVEF [27].

Similarly, pooled data from four cohort studies (*n* ≈ 6800) showed that metabolic syndrome nearly doubled the risk of early biomarker-defined cardiotoxicity—including rises in high-sensitivity troponin and natriuretic peptides—during treatment with anthracyclines or HER2-targeted agents [28]. In patients with T2DM, an individual-patient data analysis from two randomized trials demonstrated a ~1.5-fold higher incidence of clinically overt heart-failure events after anthracycline exposure [29].

Moreover, a recent multicenter registry of breast cancer survivors identified NAFLD as an independent predictor of treatment-related cardiovascular events, with adjusted hazard ratios in the range of 1.4–1.8 for HF hospitalization or premature treatment interruption [30]. Collectively, these data highlight that cardiometabolic dysfunction is not merely a background comorbidity but a quantifiable amplifier of cardiotoxic risk.

Their integration into risk-stratification algorithms (e.g., HFA/ICOS, Mayo) and follow-up intensity planning is essential to optimize prevention, guide the early use of cardioprotective therapies, and inform survivorship strategies.

In contrast to overt obesity, some patients present with sarcopenia, the loss of skeletal muscle mass and strength, either alone or in conjunction with excess adiposity (sarcopenic obesity) [27]. This condition, common in gastrointestinal cancers, older adults, and those undergoing intensive chemotherapy, is independently associated with poor cardiovascular resilience, reduced metabolic flexibility, and increased all-cause mortality [28]. Malnutrition, often overlooked, can further impair myocardial function through deficiencies in key nutrients such as selenium, thiamine, magnesium, and carnitine. Additionally, hypoalbuminemia and micronutrient deficits may exacerbate fluid retention, arrhythmogenic risk, and vulnerability to hypotensive or nephrotoxic episodes [29,30]. Beyond these pooled estimates, several large-scale observational studies reinforce the link between metabolic dysregulation and adverse cardiovascular outcomes during cancer therapy.

In a prospective cardio-oncology registry of >9000 patients, baseline insulin resistance and elevated HOMA-IR scores were independently associated with a twofold increase in cumulative incidence of subclinical LV dysfunction, as defined by ≥12% reduction in GLS during the first year of anthracycline or trastuzumab therapy [31].

A separate meta-analysis including 18 studies and >14,000 participants reported that sarcopenic obesity conferred an approximately 1.7-fold higher risk of symptomatic HF or treatment discontinuation due to cardiac events, compared with non-sarcopenic patients [32]. Notably, the pro-inflammatory phenotype associated with central adiposity appears to mediate greater troponin release and more pronounced GLS decline in patients receiving HER2-targeted therapy, as shown in a pooled biomarker-imaging substudy of three multicenter breast cancer trials [33]. These findings emphasize that refined cardiometabolic profiling, encompassing not only obesity and diabetes but also sarcopenia and inflammatory burden, should inform both baseline risk assessment and ongoing surveillance. Incorporating such evidence into practice supports timely escalation of cardioprotective measures (including early initiation of ACEi/ARB, beta-blockers, SGLT2 inhibitors, or statins where indicated) and enables individualized survivorship plans that target modifiable risk factors beyond traditional cardiovascular comorbidities.

Acute and chronic metabolic diseases, including treatment-induced hypothyroidism, hypogonadism, and electrolyte imbalances, also influence cardiovascular homeostasis. Cancer cachexia, chronic anemia, and inflammatory syndromes can increase myocardial workload and diminish functional reserve, particularly during aggressive oncologic regimens [31]. These features are often underappreciated but may be crucial in risk prediction and pre-habilitation strategies. Comprehensive patient profiling should therefore include not only conventional cardiovascular markers but also anthropometric data (BMI, waist circumference, and visceral fat estimation), body composition analysis (e.g., DEXA, bioimpedance), and laboratory evaluations including HbA1c, fasting insulin, HOMA-IR, CRP, lipid panels, liver enzymes, and nutritional markers such as prealbumin. In selected patients, imaging modalities like abdominal ultrasound for NAFLD or CT-based quantification of visceral fat may be warranted [32,33] (Table 4).

Emerging quantitative evidence supports the integration of endocrine dysfunction, cachexia, and systemic inflammation into precision risk profiling for cardio-oncology. A systematic review and meta-analysis of 11 studies (>7500 patients) found that baseline subclinical hypothyroidism was associated with a 1.4-fold increased risk of treatment-related LV dysfunction and HF hospitalization during anthracycline- or TKI-based regimens [34]. Similarly, pooled data from six cohorts of lymphoma and breast cancer patients showed that cancer cachexia and sarcopenic obesity were linked to a nearly twofold higher incidence of early treatment interruptions due to cardiovascular events [35].

In a multicenter prospective registry, patients with elevated baseline CRP (>10 mg/L) and anemia (Hb < 10 g/dL) experienced higher rates of GLS decline and troponin elevation during the first 3–6 months of cardiotoxic therapy compared with matched controls, underscoring the contribution of chronic inflammation and impaired functional reserve to early cardiac injury [36]. These data provide a compelling rationale to incorporate routine assessment of thyroid function, body composition, and inflammatory markers into cardio-oncology baseline work-ups. They also support the implementation of pre-habilitation programs—including nutritional optimization, correction of endocrine abnormalities, and early exercise interventions—to improve functional reserve before and during therapy, thereby potentially reducing both the incidence and severity of cardiotoxicity. Therefore, cardio-oncology risk stratification must evolve into a precision medicine paradigm, where traditional cardiovascular markers are enriched with detailed metabolic, endocrine, and inflammatory profiles. This holistic approach allows clinicians to proactively mitigate risk, tailor surveillance strategies, and optimize outcomes for the growing and increasingly complex population of patients with concurrent or sequential cancer and cardiovascular disease.

## 4. Multidisciplinary Care Models in Cardio-Oncology

Multidisciplinary, team-based care remains the cornerstone of high-quality cardio-oncology management, reflecting the complexity of patients who require ongoing adjustment between oncologic efficacy and cardiovascular safety [34].

Such complexity cannot be managed by a single specialty; effective care models must therefore be structured, dynamic, and collaborative [35].

The hub-and-spoke model has emerged as a proven organizational framework, particularly in regional and national healthcare systems.

In this model, tertiary or academic medical centers with advanced cardio-oncology programs (hubs) serve as centers of excellence. They develop protocols, provide specialized diagnostics—such as advanced echocardiography, strain imaging, cardiac MRI—and manage high-risk or complex patients [36,37]. Evidence supporting the hub-and-spoke approach is growing.

For example, the UK-wide ICOS-COV registry and the Italian Cardio-Oncology Network (ICOR) demonstrated that structured hub-led networks improve adherence to guideline-recommended surveillance, shorten time-to-cardiology evaluation, and reduce unnecessary treatment interruptions in cancer patients at risk of cardiotoxicity [38,39].

Similarly, a multicenter study from the US Veterans Health Administration showed that oncology patients referred early to dedicated hub-center cardio-oncology programs had lower rates of unplanned heart-failure hospitalizations and more frequent continuation of optimal cancer therapy [40]. Spoke institutions, often community or regional hospitals, deliver frontline oncology care but benefit from standardized referral pathways, shared protocols, and direct access to hub-center expertise.

Integration through shared electronic health-record (EHR) platforms, structured referral algorithms, and joint care plans ensures consistency and safety, particularly for patients in rural or underserved areas [41]. Moreover, dedicated cardio-oncology clinics form the operational backbone of this model; they typically provide structured cardiovascular assessment at three pivotal time points:

- Pre-treatment baseline risk assessment;
- Ongoing surveillance during therapy;
- Long-term survivorship follow-up.

Standardized workflows allow early detection of cardiotoxicity, prompt initiation of cardioprotective therapies, and facilitate safe resumption of oncologic treatment [42].

Joint consultation models—where cardiologists and oncologists evaluate the patient together—improve shared decision-making, patient understanding, and adherence to therapy. The extended team usually includes clinical pharmacists (to manage drug–drug interactions and polypharmacy), specialist oncology nurses and advanced nurse practitioners (for longitudinal monitoring and education), dietitians and rehabilitation experts (to optimize metabolic health and exercise capacity), and, when indicated, psychologists and social workers, particularly during survivorship phases [43]. Notably, integration with the EHR is critical: automated alerts for abnormal cardiac biomarkers or imaging findings, structured order sets, and clinical dashboards enhance communication and enable continuous quality monitoring [44].

### 4.1. Telemedicine and Digital Integration

In resource-constrained or geographically remote areas, telemedicine and virtual tumor boards have proven to be effective tools for bridging expertise gaps.

Several national-scale experiences—for example, the Brazilian Tele-Cardio-Oncology Initiative, the UK National Virtual MDT platform, and US regional tele-hub programs—have shown that virtual case reviews and remote imaging interpretation can shorten diagnostic delays, reduce travel burden, and maintain oncologic treatment intensity [45,46]. Beyond video consultations, remote biomarker sampling and cloud-based ECG or echocardiographic interpretation allow for earlier detection of subclinical cardiotoxicity, especially in patients on high-risk regimens such as anthracyclines or HER2-directed therapies.

A recent European multicenter pilot study reported that integration of tele-monitoring into cardio-oncology surveillance increased adherence to follow-up schedules by >25% and reduced unplanned hospital visits compared with conventional models [47].

However, large-scale implementation depends on interoperability between telehealth platforms and local EHRs, data-privacy safeguards, and sustainable reimbursement policies, as highlighted by both the 2022 ESC Cardio-Oncology Guidelines and ASCO survivorship frameworks [48,49].

### 4.2. Education and Research Components

Robust multidisciplinary programs also serve as educational and research hubs.

Cross-specialty training pathways for cardiologists, oncologists, and advanced practice providers, alongside joint CME initiatives, are key to workforce development.

Moreover, network-based registries and multicenter studies provide the real-world data needed to refine risk-stratification tools, test novel cardioprotective agents, and evaluate system-level interventions [50].

## 5. Monitoring and Surveillance Strategies

Cardio-oncology surveillance strategies represent a pivotal component of modern cancer care, aiming to detect cardiovascular toxicity early, ideally before irreversible myocardial injury occurs, while avoiding unnecessary interventions in low-risk individuals [46]. To achieve this, surveillance must be dynamic, personalized, and risk-adapted, incorporating the patient’s baseline cardiovascular profile, oncologic treatment plan, and comorbidities. A “one-size-fits-all” model is no longer acceptable in the context of precision medicine, especially as therapeutic agents grow in complexity and cardiotoxicity profiles diversify [47]. For anthracycline-based chemotherapy, which remains a cornerstone of treatment for breast cancer, lymphoma, and sarcomas, surveillance typically includes baseline transthoracic echocardiography (TTE) with left ventricular ejection fraction (LVEF) and global longitudinal strain (GLS), along with cardiac biomarkers such as troponin and NT-proBNP. In moderate- to high-risk patients, repeat imaging is often warranted after a cumulative anthracycline dose of 250–300 mg/m^2^ and again upon therapy completion [48]. In some cases, early troponin rise may precede echocardiographic changes, justifying the use of biomarker-driven monitoring algorithms. Dexrazoxane, a cardioprotective agent, may be considered in selected high-risk cases when cumulative dosing is unavoidable. In patients receiving HER2-targeted therapies such as trastuzumab or pertuzumab, cardiac surveillance is usually scheduled every 3 months during treatment and every 6 months during follow-up, especially when these agents are administered sequentially or concurrently with anthracyclines [49,50]. The incidence of asymptomatic LVEF decline remains significant and may require temporary treatment interruption and initiation of cardioprotective agents (e.g., beta-blockers or ACE inhibitors), particularly when GLS worsening or rising biomarkers are observed [51].

Immune checkpoint inhibitors (ICIs), now standard-of-care in multiple solid tumors, pose a unique surveillance challenge due to the rare but often fulminant nature of immune-mediated myocarditis [52]. Baseline ECG, troponin, and natriuretic peptides are recommended in all patients prior to initiation. In the first 6–12 weeks of treatment, especially in high-risk individuals or those receiving dual ICI therapy (e.g., nivolumab/ipilimumab), weekly or bi-weekly troponin monitoring has been proposed. Any symptoms or biomarker elevations warrant rapid escalation to advanced diagnostics, including cardiac MRI, PET imaging, or, in select cases, endomyocardial biopsy [52]. The window for intervention is narrow, making institutional preparedness and protocolization vital. Vascular toxicities associated with anti-VEGF agents (e.g., bevacizumab, sunitinib) and tyrosine kinase inhibitors require blood pressure monitoring at every visit and periodic assessment of renal function, electrolytes, and ECGs. For agents associated with QT prolongation (e.g., arsenic trioxide, certain TKIs), serial ECGs with corrected QT interval (QTc) measurements are essential [53]. In some cases, dose modification or replacement therapy may be necessary to mitigate arrhythmic risk.

Long-term surveillance is equally important, especially in childhood cancer survivors and young adults, who may develop late-onset cardiomyopathy or coronary artery disease decades after treatment. Guidelines increasingly recommend lifelong cardiac follow-up with periodic imaging and risk factor management, particularly in patients exposed to anthracyclines or chest irradiation [54]. The integration of advanced imaging technologies further enhances surveillance precision. Global longitudinal strain (GLS) has demonstrated superior sensitivity in detecting subclinical myocardial dysfunction compared to LVEF, and its use is rapidly expanding [55]. Moreover, 3D echocardiography, contrast-enhanced ultrasound, and cardiac MRI provide additional structural and tissue characterization, offering value in selected patients, particularly when image quality is poor or pathology is suspected [56].

Cardiac CT angiography may be indicated when vascular toxicity is a concern or in patients with overlapping ischemic symptoms. Digital health tools are increasingly transforming cardio-oncology surveillance. Wearable technologies and remote telemetry platforms now allow continuous heart rate, rhythm, and activity monitoring, facilitating early detection of arrhythmias or declining functional capacity. Some platforms integrate artificial intelligence to detect subtle changes in patterns that may precede clinical deterioration [57]. Patient-reported outcomes (PROs) collected via mobile apps or portals can identify early symptoms such as fatigue, palpitations, or dyspnea, triggering remote triage or clinic visits. Institutional integration is critical for ensuring consistency, adherence, and scalability. Embedding surveillance protocols within electronic health records (EHRs) via standardized order sets, automated reminders, and documentation templates promotes guideline adherence and multidisciplinary communication. Clinical decision support systems (CDSS) can prompt cardiology consultation when high-risk thresholds are met (e.g., rising troponin, LVEF decline, QTc > 500 ms) [58].

Quality improvement initiatives should routinely audit adherence to surveillance protocols, cardiotoxicity detection rates, and downstream outcomes to inform iterative refinements. In resource-limited settings, a tiered surveillance strategy may be necessary. Low-cost tools such as focused echocardiography and point-of-care biomarker testing can still offer substantial clinical value when integrated with clinical judgment and symptom tracking. Telemonitoring platforms may help bridge the gap by enabling real-time remote review of biomarker trends and ECG tracings by specialists based in urban centers [59]. Ultimately, surveillance in cardio-oncology must be proactive, data-driven, and integrated into both oncologic and cardiovascular care pathways. When applied appropriately, it reduces the risk of irreversible cardiac injury, supports uninterrupted cancer therapy, and improves long-term survivorship outcomes.

## 6. Healthcare System and Policy Considerations

From a healthcare systems and policy standpoint, the management of cardio-oncology patients presents a multidimensional challenge that spans clinical coordination, reimbursement structures, workforce training, and strategic planning at both national and institutional levels [60]. As the burden of cancer-related cardiovascular disease grows in parallel with improved cancer survival, the need for structured, integrated care models has become increasingly urgent. Yet, many health systems remain poorly equipped to manage this dual-morbidity population effectively and equitably [61]. One of the most pressing issues is the lack of dedicated reimbursement mechanisms for cardio-oncology services. In most countries, fee-for-service models reward procedural volume over coordinated, preventive, or longitudinal care [62]. As a result, early cardiovascular risk assessment, biomarker surveillance, long-term follow-up, and patient education, which are essential pillars of cardio-oncology, are often underfunded or excluded entirely from coverage frameworks [63]. This leads to variability in access and undermines incentives to develop proactive, cost-saving care models. Reimbursement policies must evolve to support value-based care structures that reward outcomes such as reduced cardiotoxicity incidence, hospitalization avoidance, and improved quality of life. Additionally, the absence of standardized quality indicators and performance metrics specific to cardio-oncology hinders system-level accountability and benchmarking. While metrics such as left ventricular ejection fraction decline, heart failure hospitalizations, or treatment interruptions due to cardiovascular events could be tracked, they are not yet universally codified into quality reporting systems [64]. Health authorities and professional societies must collaborate to define, validate, and implement cardio-oncology-specific KPIs that align with best practices and clinical guidelines. These metrics would allow healthcare systems to measure effectiveness, guide resource allocation, and drive continuous quality improvement [65]. Workforce development is another critical bottleneck [66]. Cardio-oncology remains an emerging field, and formalized training opportunities are limited. Most cardiologists and oncologists receive little to no structured education on managing cardiotoxicity, interpreting strain imaging or biomarkers in the oncology setting, or navigating complex decision-making when treatment goals conflict. To address this, interdisciplinary curricula must be integrated into cardiology and oncology training programs at the residency, fellowship, and continuing medical education (CME) levels [67,68].

The development of dedicated cardio-oncology fellowships, certification tracks, or dual-training modules, already underway in select academic centers, should be supported by national societies and accrediting bodies. At the policy level, health systems must recognize cardio-oncology as a strategic priority within both cardiovascular disease and cancer control programs. National cancer plans often overlook cardiovascular survivorship, while cardiac health strategies rarely account for oncologic comorbidity [69]. Policy frameworks should integrate cardio-oncology across domains such as screening guidelines, survivorship planning, electronic health record infrastructure, and population health monitoring. This integration is particularly important for aging populations, where multimorbidity is the norm and care fragmentation leads to suboptimal outcomes and increased costs.

Global organizations such as the European Society of Cardiology (ESC), the American Society of Clinical Oncology (ASCO), and the International Cardio-Oncology Society (ICOS) have begun to release consensus statements, position papers, and clinical practice recommendations to guide institutional and system-wide implementation [70,71]. However, translation of these documents into national policy and practice remains inconsistent. Countries vary widely in the availability of multidisciplinary services, funding models, and access to specialized diagnostics. Cross-border collaboration, including shared registries, training exchanges, and international summits, could accelerate standardization and knowledge transfer. Importantly, health equity must be at the center of any cardio-oncology policy discussion.

Disparities in access to care, driven by geography, socioeconomic status, race/ethnicity, and health literacy, can exacerbate outcomes in both cancer and cardiovascular disease. Underserved populations are more likely to have undiagnosed cardiometabolic risk factors, reduced access to timely diagnostics, and delayed referral to cardiology services [71,72,73]. Policymakers should consider targeted funding models, telemedicine integration, mobile care units, and community-based educational initiatives to close these gaps and ensure that cardio-oncology services are accessible to all, not just to those treated in large academic hospitals. Finally, the future of cardio-oncology policy must align with the digitization and data-driven transformation of healthcare.

Investment in real-world data infrastructure, such as multicenter registries, EHR-integrated decision support tools, and data lakes for outcomes analysis, will be crucial for both clinical optimization and health-policy refinement [74]. Data from such systems can inform evidence-based policy, support reimbursement negotiations, and guide national strategies on workforce needs, technology deployment, and quality monitoring. In conclusion, achieving sustainable, high-quality cardio-oncology care requires a comprehensive policy response that addresses infrastructure, reimbursement, education, and equity [75].

Health systems must move from reactive, fragmented approaches to a proactive, integrated model, embedding cardio-oncology within the broader architecture of cancer and cardiovascular care [76]. Only through such structural alignment can we ensure that the rapidly growing population of cancer patients and survivors receives the coordinated, preventive, and life-preserving cardiovascular care they require (Table 5).

## 7. Equity, Access, and the Future of Value-Based Cardio-Oncology

As cancer therapies prolong survival, a new challenge emerges: ensuring equitable, accessible, and value-based cardio-oncology services for all patients, regardless of socioeconomic background, geography, or healthcare system. The intersection of oncology and cardiology magnifies existing structural inequities, creating barriers to timely diagnosis, surveillance, and coordinated care [77]. These disparities contribute to widening gaps in cardiovascular outcomes among cancer patients, requiring urgent system-level responses. Health inequities in cardio-oncology are multifactorial; in fact, patients from lower-income groups, racial and ethnic minorities, rural areas, and underserved urban communities often present with undiagnosed or poorly controlled cardiovascular risk factors [78,79]. They experience delayed referrals to cardiology, limited access to diagnostic imaging, and lower participation in survivorship programs. Cultural, linguistic, and health-literacy barriers further reduce engagement and adherence [80], leading to higher rates of cardiotoxicity, treatment discontinuation, hospitalization, and mortality. Achieving equity requires intentional design and delivery of services; in fact, early outreach and screening, ideally integrated into primary care and community oncology, can identify at-risk populations [81] (Table 5). Expanding telehealth and virtual consultations reduces geographic barriers, while community health workers and patient navigators bridge communication gaps, improve follow-up, and facilitate education, transportation, and medication adherence [82,83]. Scaling equitable care demands a shift from volume-driven to value-based models. In cardio-oncology, this means prioritizing interventions that prevent treatment interruptions, reduce acute-care utilization, preserve cardiac function, and improve quality of life and cancer outcomes [84,85]. Novel approaches such as bundled payments and shared-risk contracts incentivize integrated teams to address cardiotoxicity proactively. Success depends on robust measurement tools that capture both clinical effectiveness (e.g., LVEF preservation, reduced HF admissions) and patient-centered outcomes (e.g., symptom burden, functional status, quality of life) [86] (Table 5). Digital innovation and data science will be central to advancing value-based cardio-oncology. Notably, AI- and machine-learning models are being developed to predict cardiotoxicity using EHR data, genomics, treatment regimens, and baseline comorbidities [87], enabling personalized surveillance protocols and early-intervention alerts. Digital platforms, including mobile apps, wearables, and remote monitoring, support continuous tracking of vital signs, physical activity, arrhythmias, and symptoms [88,89], reducing emergency visits, preventing progression to overt cardiac events, and enhancing patient autonomy. Real-world data registries, such as the International Cardio-Oncology Registry (ICOR), are essential for implementing and scaling value-based care. When linked to EHRs and claims data, they track outcomes longitudinally, identify care gaps, and enable benchmarking across institutions [90]. These data also inform pragmatic clinical trials, cost-effectiveness analyses, and iterative updates of guidelines to reflect diverse populations. Collaborative research networks and global partnerships further expand capacity, particularly in low- and middle-income countries, where cardio-oncology needs are rising but infrastructure remains limited [91]. Open access training platforms, remote mentorship, and cloud-based diagnostic sharing can extend expertise beyond academic centers, fostering global standards while allowing for local adaptation (Table 5). Ultimately, equity and value in cardio-oncology are strategic imperatives. Meeting the needs of the growing population of cancer survivors with cardiovascular comorbidities requires a transformation in how care is delivered, funded, and evaluated. Ensuring that all patients, regardless of geography, income, or ethnicity, have access to timely, personalized, and coordinated cardio-oncology care is essential to achieving the goal of modern medicine: not only longer survival but survival with preserved quality of life [92,93].

## 8. Discussion

**Figure 2:**
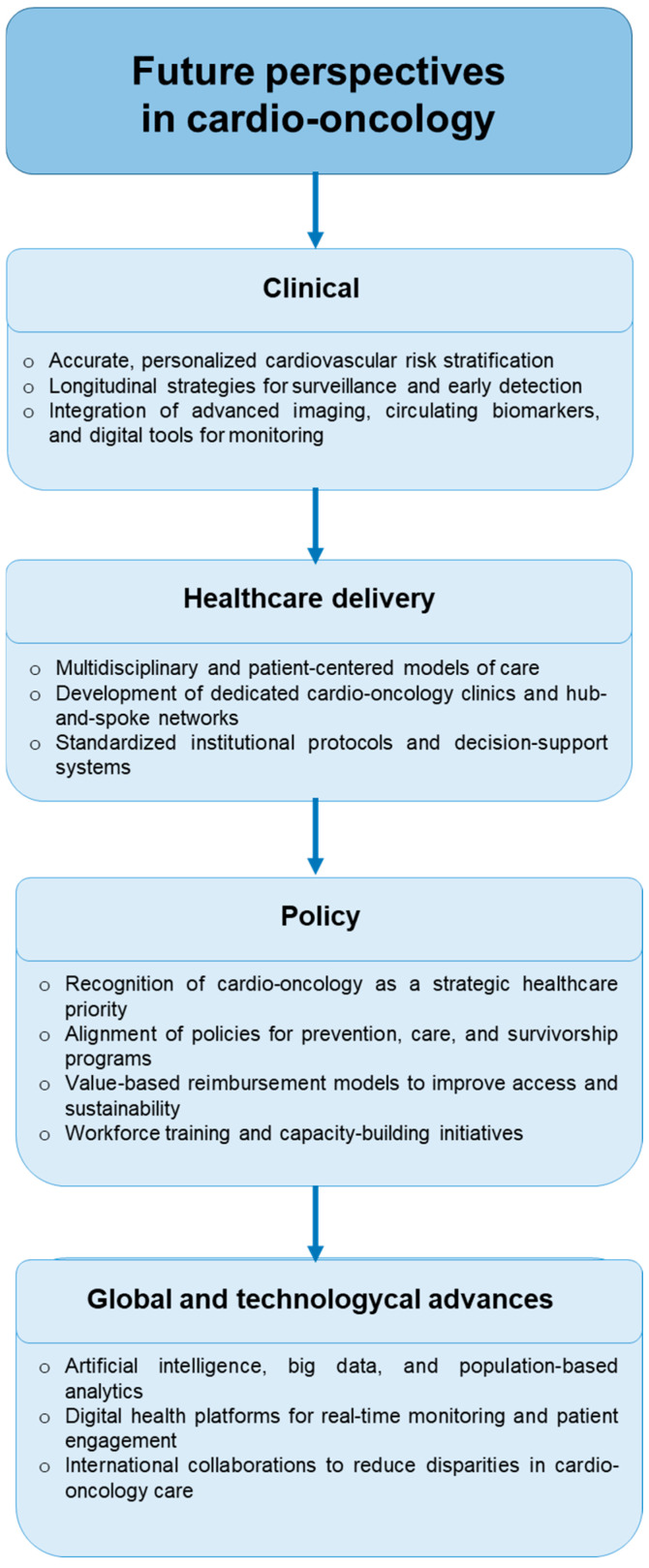
Future perspectives in cardio-oncology highlight four key domains: clinical innovation through personalized risk stratification and advanced monitoring tools; healthcare delivery via multidisciplinary care models and standardized protocols; policy initiatives that prioritize alignment, reimbursement, and workforce training; and global and technological advances, including artificial intelligence, digital health platforms, and international collaborations to reduce disparities in care.

Cardio-oncology has matured into a pivotal interface discipline in which oncologic efficacy and cardiovascular safety must be actively co-optimized rather than sequentially addressed, and the synthesis of available evidence supports a management paradigm that is simultaneously risk-adapted, biomarker- and imaging-informed, and operationalized through structured care pathways [52] (Figure 2).

Clinically, risk stratification must extend beyond traditional cardiovascular history to incorporate cardiometabolic and functional phenotyping—including visceral adiposity, insulin resistance, metabolic syndrome, NAFLD, sarcopenia, micronutrient deficits, and inflammatory burden—because these factors amplify susceptibility to anthracyclines, HER2 agents, TKIs, proteasome inhibitors, and immune-checkpoint inhibitors and modulate both the timing and the sensitivity of surveillance [63]. A practical, tiered approach pairs baseline HFA/ICOS or comparable algorithms with LVEF and GLS, high-sensitivity troponin and natriuretic peptides, ECG/QTc assessment for pro-arrhythmic therapies, and tailored surveillance intervals linked to cumulative dose and regimen (e.g., anthracyclines at dose milestones, q3-month echocardiography for HER2 therapy, front-loaded biomarker monitoring in the first 6–12 weeks of ICI exposure), with pre-defined action thresholds that trigger early cardioprotection (ACEi/ARB, beta-blockers, statins where indicated), dexrazoxane in select high-risk anthracycline use, rapid access to CMR for suspected myocarditis, and dose modification or temporary interruption under joint cardio-oncology oversight [55] (Figure 2). Survivorship is equally important, particularly after anthracyclines and chest irradiation, where lifelong atherosclerotic and heart-failure risk management, periodic imaging, and rehabilitation/exercise prescriptions reduce late events and preserve functional capacity. From a management perspective, outcomes depend on how care is organized: a hub-and-spoke network with dedicated cardio-oncology clinics, joint encounters, shared EHR pathways, and rapid diagnostics creates reliability and speed, while telemedicine, remote biomarker/ECG review, wearables, and patient-reported outcomes extend reach to community settings and shorten time to intervention (Figure 2). Hospital and system leaders should formalize this model via service-line governance, cross-specialty staffing (cardiology, oncology, pharmacy, nursing, nutrition, rehabilitation, psycho-oncology), standardized order sets and alerts embedded in the EHR, and capacity planning for imaging and day-hospital biomarker workflows; concurrently, quality is driven by cardio-oncology-specific KPIs—including rates of LVEF decline, treatment interruption attributable to CV events, HF hospitalizations, time from signal to cardiology evaluation, adherence to surveillance schedules, and return-to-therapy after toxicity—and by routine multidisciplinary morbidity and utilization reviews [91]. Financial sustainability requires transitioning from fee-for-service to value-based constructs (bundles/shared-risk) that reward prevention of cardiotoxicity and avoidance of admissions; real-world data registries linked to clinical and claims datasets are essential to benchmark performance, refine risk models with AI-enabled prediction, and negotiate reimbursement aligned to measurable outcomes [94] (Figure 2). Finally, equity must be engineered into the pathway, with proactive screening in primary care and community oncology, navigators for low-literacy or resource-limited patients, mobile diagnostics, and virtual tumor boards, because without deliberate design, the benefits of modern cardio-oncology remain concentrated in tertiary centers. In sum, the clinical science mandates precision risk assessment and early intervention, while the management science demands standardized, networked, data-driven delivery that maintains oncologic intensity, minimizes cardiovascular harm, and scales equitably across health systems [94]. Although digital health innovations, including telemedicine, remote biomarker and ECG monitoring, wearables, and AI-driven risk prediction tools, have expanded the reach of cardio-oncology services, they present practical, ethical, and systemic challenges that must be addressed for equitable and sustainable implementation. Persistent data privacy and security concerns, along with varying regulatory requirements (e.g., GDPR, HIPAA), often hinder the seamless sharing of clinical and imaging data across institutions and jurisdictions. Moreover, the lack of interoperability between digital platforms and electronic health records (EHRs) limits the integration of these technologies into routine workflows, reducing their impact on timely decision-making. Adoption is further limited in low- and middle-income countries (LMICs) and rural areas of high-income countries due to insufficient broadband access, limited technological infrastructure, and gaps in digital literacy among patients and providers. Without deliberate planning and targeted investment, these disparities risk widening the cardio-oncology care gap, undermining the promise of technology-enabled equity. Addressing these barriers requires policy incentives, scalable low-cost solutions, capacity building, and public–private partnerships to ensure that innovation benefits not only tertiary centers but also community settings. The transition to value-based care models, which aim to reward prevention of cardiotoxicity, preservation of cardiac function, and avoidance of treatment interruptions, also faces implementation barriers. Challenges include the absence of universally accepted cardio-oncology quality metrics, the fragmentation of reimbursement systems, and the lack of integrated longitudinal outcome data required to benchmark performance and negotiate payment based on results. Furthermore, the increasing use of AI-assisted clinical decision-making introduces issues of algorithm transparency, accountability, and regulatory oversight, as clinicians remain ultimately responsible for patient outcomes. These challenges highlight that technology and innovative financing models are not panaceas; their success depends on robust clinical governance, multidisciplinary workforce development, and supportive infrastructure that allow emerging tools to be translated into safe, effective, and equitable cardio-oncology care.

## 9. Limitations and Clinical Perspectives

This review is narrative in design. Although we applied a structured search strategy and qualitatively appraised key risk-of-bias domains (patient selection, outcome ascertainment, and confounding adjustment), the absence of a formal PRISMA workflow, pooled effect estimates, and quantitative heterogeneity assessment limits definitive comparative inferences. The breadth and heterogeneity of the evidence, spanning randomized trials, observational cohorts, registries, organizational models, and policy analyses, precluded meta-analytic synthesis but enabled a practice-oriented overview. Beyond these methodological aspects, several conceptual limitations should be acknowledged. The framework we present integrates clinical, imaging, and biomarker domains to guide cardio-oncology decision-making; however, it does not constitute a rigid, quantitatively validated scoring system. This choice was deliberate, given the heterogeneity of cancer populations, therapeutic exposures, and healthcare settings, and the scope of a narrative, clinically oriented review. Existing validated scores (e.g., HFA–ICOS; general CV risk engines such as SCORE2) offer useful anchors yet display restricted generalizability across diverse tumor types, treatment combinations (including novel targeted and immune therapies), and real-world contexts.

To strengthen the logical consistency and practical applicability of the proposed model, we outline a semi-quantitative, domain-based framework intended as a conceptual proposal rather than a validated scoring system (Table 6 and Table 7).

This framework is designed to assist clinicians in stratifying cardiovascular risk among cancer patients and survivors by integrating three complementary domains: baseline cardiovascular phenotype, cancer-therapy exposure, and early treatment-related dynamics. It is deliberately simple and adaptable across tumor types and clinical settings.

### 9.1. Baseline Cardiovascular Phenotype

This domain captures the patient’s pre-treatment cardiovascular and cardiometabolic status. It includes prior cardiovascular disease (myocardial infarction, heart failure, valvular disease, cardiomyopathy, arrhythmia), together with cardiometabolic comorbidities such as hypertension, diabetes or hyperglycemia, obesity and central adiposity, sarcopenia or sarcopenic obesity, MASLD (metabolic dysfunction-associated steatotic liver disease), dyslipidemia, and chronic kidney disease. Functional capacity and frailty indicators—performance status, inflammatory markers, anemia, or low physiologic reserve—should also be considered, as they increase vulnerability to cardiotoxicity.

### 9.2. Cancer-Therapy Exposure and Intensity

This domain reflects the intrinsic cardiotoxic potential of anticancer regimens and the patient’s cumulative treatment burden. Relevant components include anthracycline cumulative dose, sequential or concomitant HER2-targeted therapy, use of VEGF/VEGFR tyrosine-kinase inhibitors, BCR-ABL inhibitors, proteasome inhibitors, immune checkpoint inhibitors (single or dual), and hormonal therapies with known cardiovascular impact. Radiation exposure (mean heart dose, left-sided fields) and the overall complexity of prior and concurrent treatments (pluri-treated patients, high cumulative dose intensity, or polypharmacy) are additional determinants.

### 9.3. Early Treatment-Related Dynamics

This domain encompasses on-treatment markers that signal emerging risk, such as serial biomarker trends (high-sensitivity troponin, NT-proBNP), echocardiographic parameters (LVEF trajectory, global longitudinal strain—GLS), and electrocardiographic findings (QTc prolongation, new arrhythmias, or conduction delays). Moreover, dynamic changes in these parameters provide actionable early warnings of subclinical injury and help refine individual risk levels (Table 6).

**Table 6:** *Proposed semi-quantitative framework for cardiovascular risk stratification in cardio-oncology.* This conceptual matrix integrates three major domains, namely baseline cardiovascular phenotype, cancer-therapy exposure, and early treatment-related dynamics, each scored from 0 (minimal risk) to 3 (severe risk). The cumulative score (0–9) allows a structured, semi-quantitative categorization of patients into low, intermediate, high, and very-high risk strata. The proposed weighting and thresholds are illustrative and intended for conceptual use only; prospective calibration and validation are required before clinical implementation. ↓: decrease.

Domain	0 (Minimal)	1 (Mild)	2 (Moderate)	3 (Severe)
**Baseline cardiovascular phenotype**	No CVD, ≤1 controlled risk factor	≥2 risk factors, mild obesity	Obesity + MASLD or sarcopenia; early CKD	Prior CVD (CAD/HF/valvular), advanced CKD, frailty/inflammatory state
**Therapy exposure & intensity**	Low-risk regimens, no radiation	Single moderate-risk drug or low radiation dose	High-risk single agent (anthracycline at dose milestone, HER2 after anthracycline, VEGF-TKI)	Combination/high-intensity regimen (dual ICI, anthracycline + HER2, multiple prior lines, high MHD)
**Early dynamics (biomarkers/imaging/ECG)**	Stable biomarkers and imaging	Borderline biomarker rise or minor GLS change	Persistent troponin/BNP elevation or GLS ↓10–15%	GLS ↓ ≥ 15%, LVEF ↓ ≥ 10 points < 50%, marked QTc ≥500 ms, new arrhythmia

Each domain is assigned 0–3 points, generating a composite score from 0 to 9 that stratifies patients into practical risk categories (Table 7):

**Table 7:** *Illustrative risk categories and corresponding clinical management strategies*. Suggested management pathways reflect increasing surveillance intensity and cardioprotective intervention according to cumulative risk burden. The framework is intended to complement, not replace, validated risk assessment tools (e.g., HFA–ICOS, SCORE2) and should be adapted to institutional resources and patient-specific factors.

Total Score	Risk Level	Suggested Clinical Approach
**0–2**	Low	Routine surveillance per guidelines; lifestyle optimization
**3–5**	Intermediate	Shorter intervals for echocardiography/biomarker monitoring; early cardioprotection (ACEi/ARB, β-blocker, statin as indicated)
**6–7**	High	Structured cardio-oncology co-management; proactive cardioprotective therapy; lower thresholds for imaging escalation; consider dexrazoxane if anthracycline required
**8–9**	Very-high	Multidisciplinary discussion; potential treatment modification; close telemetry during high-risk therapy; rapid CMR access for suspected myocarditis or acute toxicity

Notably, this semi-quantitative matrix does not replace validated tools; it organizes the multi-domain signals already used in practice—making explicit how cardiometabolic state (obesity/central adiposity, insulin resistance/diabetes, MASLD, sarcopenia), prior CVD, treatment complexity (multi-treated patients), and early biomarker/imaging changes jointly shape risk. Threshold examples (e.g., GLS ↓ ≥ 15%, LVEF ↓ ≥ 10 points to <50%, marked QTc prolongation, persistent hs-troponin/NT-proBNP elevation) align with widely adopted clinical signals; precise weights and cut points require prospective calibration and external validation across tumor types, regimens, and health-system settings. Integration with EHR-embedded pathways and registries will be essential to assess discrimination, calibration, actionability, and impact on patient-centered outcomes.

Finally, as with any narrative synthesis, risks of selective reporting, publication bias, and expert interpretation cannot be excluded. Future work should (i) calibrate and validate the proposed matrix in prospective cohorts, (ii) test augmentation with emerging features (e.g., genomics, metabolomics, AI-derived imaging metrics), and (iii) quantify the effect of risk-tier-linked interventions on treatment continuity, HF hospitalizations, and survival.

## 10. Conclusions

Cardio-oncology has evolved from a subspecialty focused primarily on detecting chemotherapy-induced left-ventricular dysfunction to a broader discipline that aims to anticipate, prevent, and manage cardiovascular risk throughout the cancer journey. The evidence reviewed here highlights three priorities for translating knowledge into improved outcomes. First, risk stratification must become precise and multidimensional, integrating baseline cardiovascular comorbidities with metabolic, functional, and treatment-related factors, including cumulative exposure to anthracyclines, HER2-targeted agents, and thoracic radiation. Dynamic monitoring with biomarkers and imaging should allow re-stratification over time and trigger timely cardioprotective interventions. Second, effective care delivery depends on robust multidisciplinary networks, ideally organized in hub-and-spoke models that embed cardio-oncology services within cancer centers and community settings. Adoption of digital tools and telemedicine can reduce inequities in access, enhance continuity of care, and facilitate early intervention when subclinical injury is detected. Third, long-term sustainability requires alignment of reimbursement, policy, and quality metrics with value-based objectives. Incentives that reward prevention of treatment interruptions, preservation of cardiac function, and better survivorship outcomes are essential to embed cardio-oncology into standard cancer care. Taken together, these elements define cardio-oncology as an integrated, proactive, and equity-oriented field that bridges cardiology, oncology, primary care, and health-policy domains. Ongoing research, spanning novel biomarkers, personalized cardioprotective therapies, pragmatic trials of care models, and real-world data infrastructure, will further refine this approach and support the transition from reactive treatment of cardiotoxicity to anticipatory, patient-centered cardiovascular care for all individuals living with and beyond cancer.

## Figures and Tables

**Table 1:** The table summarizes key drug classes and thoracic radiation associated with cardiovascular complications, highlighting the predominant toxicity patterns supported by evidence from recent guidelines and meta-analyses.

Anticancer Therapy	Representative Agents	Main Cardiovascular Toxicities
Anthracyclines	Doxorubicin, Epirubicin	Dose-dependent LV dysfunction and HF [1,3,11]
Platinum-based Compounds	Cisplatin	Vascular toxicity: hypertension, thromboembolism, premature atherosclerosis [4]
Alkylating Agents	Cyclophosphamide	Acute HF at high dose; endothelial/vascular injury similar to platinum agents [4]
HER2-Targeted Therapies	Trastuzumab, Pertuzumab	Usually reversible LV dysfunction, especially with prior anthracyclines [5,11]
VEGF-Pathway Inhibitors	Bevacizumab, Sunitinib, Sorafenib	Hypertension, thromboembolic events, HF due to endothelial dysfunction [6]
BCR-ABL Tyrosine Kinase Inhibitors	Imatinib, Dasatinib, Ponatinib	Pulmonary hypertension, arterial occlusion, arrhythmias [7]
Proteasome Inhibitors	Bortezomib, Carfilzomib	Hypertension, thromboembolic/ischemic events, LV dysfunction [8]
Immunomodulatory Drugs	Thalidomide, Lenalidomide	Increased risk of venous/arterial thromboembolism [8]
Immune-Checkpoint Inhibitors	Nivolumab, Pembrolizumab, Ipilimumab	Immune-mediated myocarditis, pericarditis, vasculitis—potentially fulminant [9]
Hormonal Therapies	Aromatase inhibitors, Androgen-deprivation therapy (ADT)	Increased risk of ischemic heart disease and/or HF in predisposed/older patients [10]
Thoracic/Breast Radiation)	Adjuvant or palliative chest/breast RT	Late-onset CAD, valvular disease, restrictive cardiomyopathy [11]

**Table 2:** This table outlines key healthcare strategies and system-level approaches for delivering integrated cardio-oncology care.

Healthcare Strategy	Key Features	Supporting References
**Multidisciplinary Care Model**	Collaboration among cardiologists, oncologists, internists, radiologists, specialist nurses, pharmacists, rehabilitation experts, and patient navigators to ensure coordinated and patient-centered care.	[12,13,14,15]
**Dedicated Cardio-Oncology Units**	Units embedded within cancer centers or cardiology institutions, enabling protocolized care pathways for baseline risk stratification, continuous surveillance during treatment, early management of cardiotoxicity, and structured long-term follow-up.	[16,17,18]
**Monitoring Approaches**	Use of echocardiography (LVEF, GLS), ECG, troponin/BNP assays, and advanced imaging (CMR as appropriate), applied with risk-adapted surveillance schedules derived from ESC/ICOS recommendations.	[3,11,19,20,21]
**Health** **-System and Policy Strategies**	Support for integrated care pathways, implementation of standardized protocols and quality metrics, promotion of equitable access, expansion of telemedicine and digital-health tools, and development of policy and reimbursement frameworks for prevention and survivorship care.	[22,23,24,25,26]

**Table 3:** The table summarizes the structured—but non-systematic—search strategy adopted to guide the narrative synthesis. It specifies the databases, time frame, key search terms, and inclusion/exclusion criteria used to identify relevant literature on cardiotoxicity, risk stratification, management models, and health-policy issues in cardio-oncology. This approach ensured transparency in source selection while avoiding the formal constraints of a PRISMA-based systematic review, which was deemed inappropriate given the heterogeneity of study types and the focus on clinically oriented management and policy perspectives.

Parameter	Details
Databases searched	PubMed/MEDLINE, EMBASE
Time frame	January 2010–September 2025
Language	English
Search terms/keywords	“cardio-oncology”, “cardiotoxicity”, “risk stratification”, “biomarkers”, “imaging”, “multidisciplinary care”, “hub-and-spoke”, “digital health”, “value-based care”, “health disparities”, “survivorship”
Boolean operators	Combinations of AND/OR without truncation to improve retrieval
Inclusion criteria	Clinical studies (RCTs, cohorts, registries), meta-analyses, guidelines/position papers, health policy, and organizational reports relevant to cardio-oncology
Exclusion criteria	Narrative reviews, editorials, commentaries, case reports, pre-clinical experimental studies without direct clinical implications, conference abstracts lacking full data
Additional sources	Reference lists of included studies, relevant ESC, ASCO, ICOS guidelines, and consensus statements

**Table 4:** Summary of major domains contributing to cardio-oncology risk assessment and their integration into validated tools, including the HFA/ICOS risk score and the Mayo Clinic algorithm. ↓: decrease.

Risk Domain	Key Components/Suggested Measures	Integration into Risk Model & Clinical Implications
**Baseline Cardiovascular Risk**	Hypertension, dyslipidemia, prior CAD, prior HF, valvular or congenital heart disease; smoking; CKD	Provides the baseline layer for risk stratification; incorporated in HFA/ICOS and Mayo scores; identifies patients needing intensified baseline imaging and closer follow-up.
**Oncologic Treatment Exposure**	Type and cumulative dose of anthracyclines, HER2-targeted agents, VEGF/VEGFR-TKIs, BCR-ABL TKIs, ICIs; radiation to chest/breast (dose, field)	Determines drug- or modality-specific risk tier; drives schedule of biomarker/echo surveillance and need for early cardioprotection (e.g., dexrazoxane for anthracyclines).
**Validated Risk Scores**	HFA/ICOS risk score, Mayo Clinic algorithm (baseline CV + therapy + age + imaging)	Provides initial patient classification (low/intermediate/high/very-high); serves as the backbone of the model.
**Biomarkers & Cardiac Imaging**	High-sensitivity troponin, NT-proBNP, LVEF, GLS, ECG, ± cardiac MRI	Supplies dynamic re-stratification during therapy; triggers escalation if GLS ↓ ≥ 15% or LVEF ↓ ≥ 10 pts to <50%, or persistent biomarker elevation.
**Metabolic & Body-Composition Profile**	Visceral obesity (waist circumference, VAT imaging), insulin resistance/HOMA-IR, metabolic syndrome	Functions as a risk modifier that amplifies baseline/therapy-related risk and increases cardiotoxicity; targeted interventions (weight loss, SGLT2i, GLP-1 RA).
**Diabetes/Hyperglycemia**	T2DM, impaired fasting glucose, or HbA1c elevation	Contributes to microvascular and mitochondrial vulnerability; intensifies need for cardioprotective agents (ACEi/ARB, SGLT2i) and glycemic control.
**NAFLD/Liver Dysfunction**	Steatosis or steatohepatitis (imaging, liver stiffness), abnormal LFTs	Adds systemic inflammatory and pharmacokinetic burden; considered a risk enhancer for anthracycline and ICI toxicity.
**Sarcopenia & Nutritional Status**	Low skeletal-muscle mass or sarcopenic obesity (CT- or DEXA-based), hypoalbuminemia, micronutrient deficiencies	Reflects low physiologic reserve and higher vulnerability to HF and treatment complications; prompts tailored nutritional and exercise interventions.
**Endocrine/Hormonal Disorders**	Hypothyroidism, hypogonadism, cortisol excess/deficiency, electrolyte imbalance	Can be treatment-related or chronic; alters hemodynamics and recovery; needs to be corrected to optimize CV and cancer outcomes.
**Functional/Inflammatory Reserve**	Cachexia, anemia, elevated CRP/IL-6, cytokine burden	Predicts intolerance to aggressive therapy and poorer LV recovery; motivates early introduction of protective strategies.
**Comprehensive Assessment Tools**	Anthropometrics (BMI, waist circumference), body composition (DEXA, bio-impedance), routine metabolic & inflammatory labs	Provides a structured, semi-quantitative profile that complements validated scores and allows personalized surveillance and tailored interventions.

**Table 5:** This table outlines the systemic, financial, educational, technological, and equity-driven factors essential for sustainable, high-quality cardio-oncology care. It highlights challenges and actionable strategies at the interface of healthcare delivery and policy.

Domain	Key Issues	Implications/Strategic Solutions	References
Care Coordination	Fragmented services across cardiology and oncology	Develop integrated, multidisciplinary care models embedded within cancer and cardiac care systems	[34,35,36]
Reimbursement Models	Fee-for-service disincentivizes preventive, longitudinal care	Shift to value-based reimbursement models (e.g., bundled payments, shared-risk contracts) that reward quality and outcomes	[37,38]
Access and Equity	Disparities by income, race/ethnicity, geography, and health literacy	Expand telemedicine, mobile clinics, community health-worker programs, and patient-navigator services to reach underserved populations	[39,40]
Workforce Development	Lack of formal training in cardio-oncology for most clinicians	Create interdisciplinary training tracks, fellowships, and CME programs in cardio-oncology	[41]
Quality Metrics	Absence of standardized KPIs or reporting frameworks	Establish cardio-oncology-specific quality indicators (e.g., rates of LVEF decline, HF hospitalization, cancer-therapy discontinuation)	[42]
Health-Policy Integration	Cancer and cardiac strategies remain siloed; survivorship often neglected	Integrate cardio-oncology into national cancer and cardiovascular health plans, EHR-based care pathways, and clinical guidelines	[43,44]
Global Standardization	Wide variation in services, diagnostics, and infrastructure	Promote international collaboration, registry-based benchmarking (e.g., ICOS/ICOR), global training platforms, and summit-driven knowledge exchange	[44,45]
Data and Digital Infrastructure	Lack of real-world outcome tracking and clinical-decision support	Invest in disease-specific registries (e.g., ICOR), interoperable EHRs, AI-driven decision support, and remote-monitoring systems	[44,45]
Technology and Innovation	Limited use of predictive analytics and patient-engagement tools	Leverage AI, wearable sensors, mobile apps, and tele-monitoring platforms to personalize care and prevent adverse events	[44,45]
Value-Based Care	Persistent focus on volume-based delivery	Prioritize care models and interventions that reduce hospitalizations, preserve cardiac function, and improve quality of life	[37,38,45]

## Data Availability

No new data were created or analyzed in this study.

## Abbreviations

The following abbreviations are used in this manuscript:

ACEi	Angiotensin-Converting Enzyme inhibitor
ADT	Androgen Deprivation Therapy
AI	Artificial Intelligence
ARB	Angiotensin II Receptor Blocker
ASCO	American Society of Clinical Oncology
BMI	Body Mass Index
BNP	B-type Natriuretic Peptide
BP	Blood Pressure
CAD	Coronary Artery Disease
CDSS	Clinical Decision Support System
CMR	Cardiac Magnetic Resonance
CRC	Colorectal Cancer
CRP	C-reactive Protein
CT	Computed Tomography
CTCA/CCTA	Coronary CT Angiography
CVD	Cardiovascular Disease
DEXA	Dual-Energy X-ray Absorptiometry
ECG	Electrocardiogram
EHR	Electronic Health Record
ESC	European Society of Cardiology
GLS	Global Longitudinal Strain
HF	Heart Failure
HFpEF	Heart Failure with preserved Ejection Fraction
HFrEF	Heart Failure with reduced Ejection Fraction
HFA	Heart Failure Association (of the ESC)
HDL	High-Density Lipoprotein
HER2	Human Epidermal Growth Factor Receptor 2
HOMA-IR	Homeostatic Model Assessment of Insulin Resistance
HTN	Hypertension
ICI	Immune Checkpoint Inhibitor
ICOR	International Cardio-Oncology Registry
ICOS	International Cardio-Oncology Society
KPI	Key Performance Indicator
LDL	Low-Density Lipoprotein
LV	Left Ventricle/Left Ventricular
LVEF	Left Ventricular Ejection Fraction
MetS	Metabolic Syndrome
MRI	Magnetic Resonance Imaging
NAFLD	Non-Alcoholic Fatty Liver Disease
NT-proBNP	N-terminal pro-B-type Natriuretic Peptide
NYHA	New York Heart Association (functional class)
PCI	Percutaneous Coronary Intervention
PET	Positron Emission Tomography
PRO	Patient-Reported Outcome
QoL	Quality of Life
QTc	Heart-rate–corrected QT interval
RCT	Randomized Controlled Trial
RPM	Remote Patient Monitoring
T2DM	Type 2 Diabetes Mellitus
TKI	Tyrosine Kinase Inhibitor
TTE	Transthoracic Echocardiography
VEGF	Vascular Endothelial Growth Factor
VTE	Venous Thromboembolism
